# Non-Destructive Evaluation of Mortar with Ground Granulated Blast Furnace Slag Blended Cement Using Ultrasonic Pulse Velocity

**DOI:** 10.3390/ma15196957

**Published:** 2022-10-07

**Authors:** Chi Kang Loke, Barry Lehane, Farhad Aslani, Subhra Majhi, Abhijit Mukherjee

**Affiliations:** 1 Department of Civil, Environmental and Mining Engineering, School of Engineering, The University of Western Australia, Crawley, WA 6009, Australia; 2 School of Engineering, Edith Cowan University, Joondalup, WA 6027, Australia; 3 School of Civil and Mechanical Engineering, Bentley, WA 6012, Australia

**Keywords:** non-destructive testing, ultrasonic pulse velocity, mortar, slag, water-to-cement, compressive strength

## Abstract

Non-destructive evaluation using ultrasonic pulse velocity (*V_p_*) testing has extensive applications in the concrete industry. With advances in construction technology, the use of ground granulated blast furnace slag (GGBFS) as a partial replacement to cement in a concrete mix is growing in popularity primarily because it reduces the initial capital cost of raw materials and the associated energy costs. This paper investigates the effect of the water-to-cement (*w_c_*) ratio and the cement content replaced by GGBFS on the development with time of the ultimate compressive strength ($f_{c}^{\prime }$) and the compression wave velocity (*V_p_*) of mortar. The results showed that in the case of mortar with higher percentages of GGBFS replacement (where nucleation surfaces are more abundant), increasing *w_c_* can increase $f_{c}^{\prime }$ but cause a decrease in *V_p_*. The posterior hydration process is highly dependent upon the water particles in the mixture after the first stage of hydration. After 7 days of curing, experimental results show that the $f_{c}^{\prime }$ of slag blended cement mix design *w_c_* ratio of 0.6 surpassed the $f_{c}^{\prime }$ value of an Ordinary Portland cement. A regression model correlating the $f_{c}^{\prime }$ and *V_p_* of slag blended mortar is developed, which can be used to predict $f_{c}^{\prime }$ at concrete ages ranging from 1 day to 28 days for mixes with GGBFS percentage replacement values ranging from 15% to 45%.

## 1. Introduction

Although concrete is a primary material in construction, increasing world demands have led to an increase in cement production and associated undesirable increases in carbon dioxide emissions. The incorporation of a range of recyclable materials or by-products has consequently become a primary focus in the industry as these materials can be used as partial replacements to cement in concrete production and hence provide positive environmental, economic and technological benefits.

One of the common by-products that can be used as cement substitution is ground granulated blast furnace slag (GGBFS). GGBFS is a by-product of the smelting process used to convert iron ore to pig iron. It is highly cementitious with the potential to improve the strength and durability of the concrete [1,2,3,4]. However, the heterogeneity and properties of concrete are strongly governed by the mixture composition and the hydration process. This reaction between water and the materials used in the design mix significantly influences compressive strength development [1,4].

GGBFS is finer than Ordinary Portland cement (OPC) and leads to a less porous structure with fewer capillary pores. Consequently, there is increased durability due to a denser microstructure with a finer distribution of pores however this is strongly governed by the degree of hydration. The interaction between GGBFS and cement grains at a microscopic level was examined by Escalante-García and Sharp [5], they indicated that the pozzolanic reaction rate is directly proportional to the particle size of replacement materials, and the effect of additional water available during the later stages of hydration.

Numerous studies investigated different aspects of slag blended mortars or concrete in terms of fresh and mechanical properties [6]. These studies have aimed to determine the optimal replacement percentage of OPC by GGBFS, while achieving the same or greater compressive strength. Many of these studies support the findings obtained in the investigation of Tasdemir et al. [7] who found that slag blended concretes tend to have slower compressive strength gain development than OPC concretes but may have similar or higher strength than OPC concretes at later ages.

Compression wave velocity (*V_p_*) is frequently measured in ultrasonic pulse velocity (UPV) testing of concrete to evaluate and monitor the mechanical properties of concrete. Many empirical equations relating *V_p_* with $f_{c}^{\prime }$ of a range of different concrete types have been established; a selection of reported relationships is summarised in Table 1. Najim et al. [8] suggested a linear relationship between *V_p_* and $f_{c}^{\prime }$ while others involve higher order polynomials [9], logarithmic [10] or multivariable regression equations [11,12,13,14,15]. However, an exponential format is the most popular form, as presented in [3,11,16,17,18,19].

Demirboga et al. [3] reported that the *V_p_* of concrete containing 60% and 70% of GGBFS was lower than OPC concrete. They also found that concrete with 50% GGBFS replacement has a relatively low early-age compressive strength but a strength comparable to OPC concrete at later ages. The investigation carried out by Turkmen et al. [19] showed that slag blended concrete with lower GGBFS replacement proportions of between 10% and 30% achieved approximately the same or greater compressive strength than OPC concrete after 28 days. These findings are also supported by Le et al. [18] with 30% of GGBFS replacement. All these studies confirmed that the concrete mixture composition has a variety of influences on $f_{c}^{\prime }$ and *V_p_*.

It is evident from Table 1 that most of the established empirical correlations between $f_{c}^{\prime }$ and *V_p_* are single parameter equations. In this study, different regression methods were utilised to predict the $f_{c}^{\prime }$ of 9 mortar mixes with slag blended cement and with GGBFS replacement proportions of 15%, 30% and 45%. In addition to that, *V_p_* measurements are obtained to profile the impact on $f_{c}^{\prime }$ for mortars with varying degrees of GGBFS replacement. The test results were used to develop a strength prediction model which describes $f_{c}^{\prime }$ as a function of the pulse velocity (*V_p_*), concrete age (*d*) and water-to-cement ratio (*w_c_*).

## 2. Materials and Methods

### 2.1. Materials

Ordinary Portland cement (OPC) was used for all the mixes examined in the present study, while 45/50 grade silica sand was employed as the natural fine aggregate. The GGBFS supplied by Australian Steel Mill Services Pty Ltd. (Bassendean, Australia) was used as a replacement for OPC. The chemical composition and physical properties of these constituents are summarised in Table 2, Table 3 and Table 4.

### 2.2. Mix Designs

In this case, 12 mortar mixtures, with proportion details listed in Table 5, were prepared for the investigation of the slag blended mortar. It is seen that the ratio of sand in the natural fine aggregate was fixed at twice the combined weight of OPC and GGBFS in all samples. Three different *w_c_* ratios were considered (0.4, 0.5 and 0.6). Additionally, three cement replacement percentages with GGBFS at levels of 15%, 30%, and 45% by the total cement weight were investigated. OPC mixes—CM-1, CM-2, and CM-3 were used to illustrate the influence of $w_{c}$ without any GGBFS. Superplasticizer (MasterEase3000 by BASF, Ludwigshafen, Germany) was also used to maintain adequate workability. The mortar mixtures were typically mixed for 5 to 7 mins before placement in cubic 100 mm wide moulds. These specimens were stored and cured at a temperature of 25 °C and humidity above 80%.

### 2.3. Testing

Compressive strength tests were performed (using a Baldwin universal testing machine) on cube samples in accordance with ASTM C109-21 [21] after curing periods of 1, 4, 7, 14 and 28 days. Tests were performed on three specimens for each mix at a constant displacement rate of 0.5 mm/min.

An ultrasonic instrument developed and manufactured by Proceq, PUNDIT Lab+, (Schwerzenbach, Switzerland) was used to obtain measurements of *V_p_*. The testing complied with several established standards such as ASTM C597-02 [22], BS 1881-203:1986 [23], CECS 21-2000 [24], BS EN 12504-4:2004 [25], ISI 13311-1:1992 [26], and ISO 1920-7:2004 [27]. The direct transmission method employed for this study involved a transducer pair (emitter and receiver) placed on opposite faces of the cubic specimens. During an ultrasonic measurement, a square-wave signal with 500 V excitation voltage and a pulse width of 9.3 μm and a nominal frequency of 54 kHz was generated by the PUNDIT Lab+ at a sampling rate of 2 MHz. Measurements of *V_p_* were obtained for all specimens prior to compressive strength tests.

## 3. Test Results

### 3.1. Mechanical Compressive Strength

#### 3.1.1. Influence of GGBFS as a Partial Replacement of Cement on Compressive Strength

**Figure 1:**
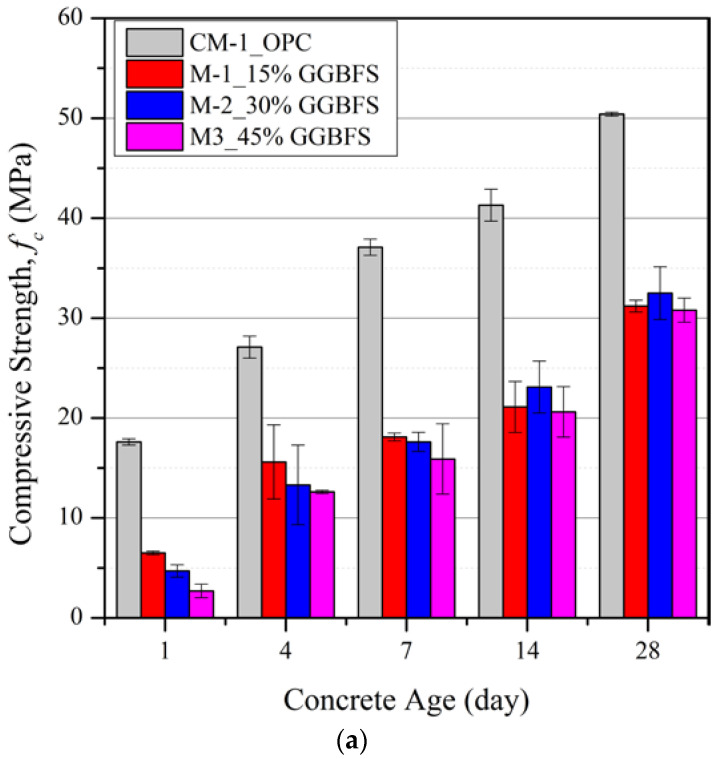

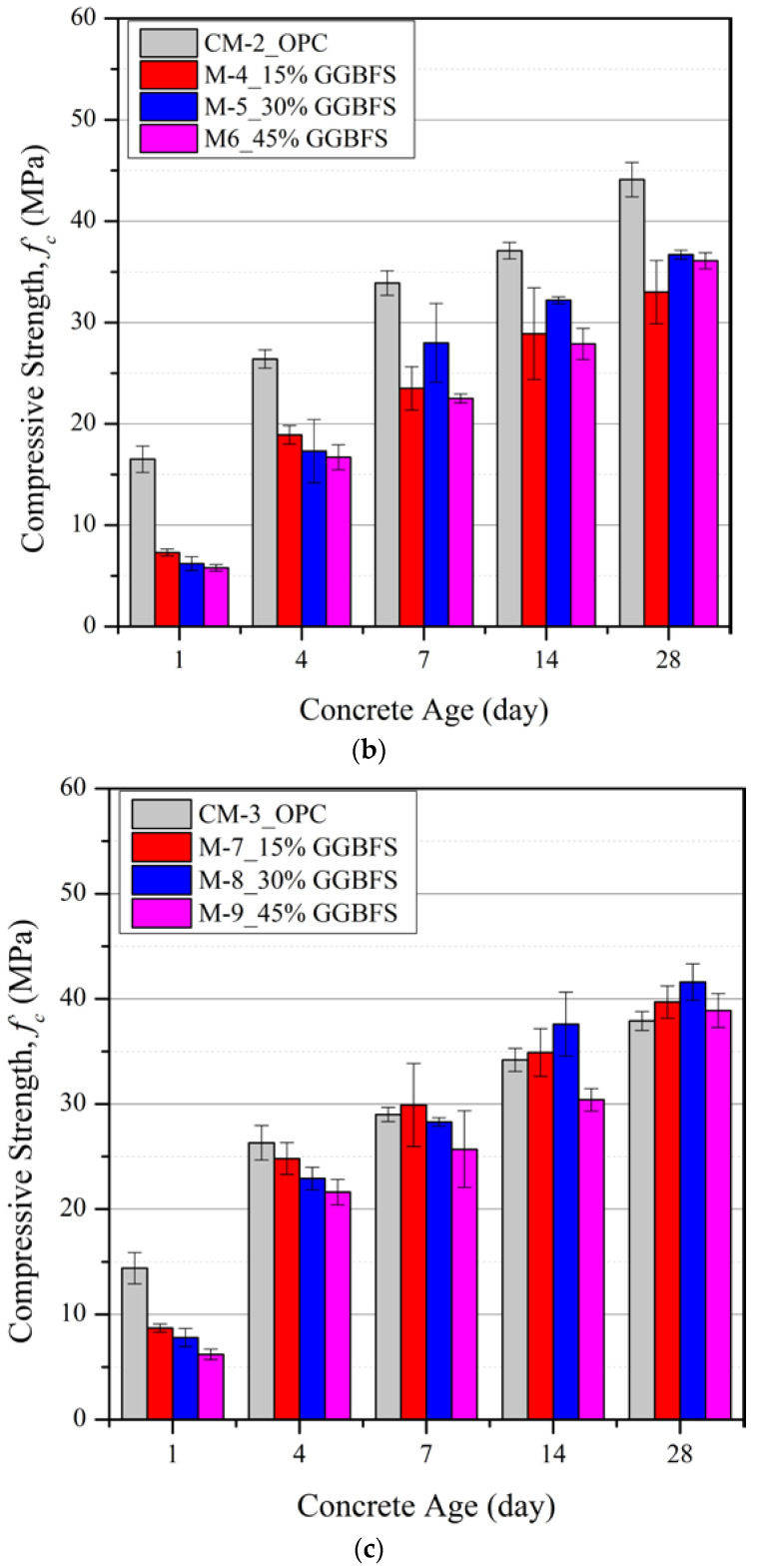
Concrete strength development of mortar mixtures with: (**a**) *w_c_* = 0.4, (**b**) *w_c_* = 0.5 and (**c**) *w_c_* = 0.6.

Figure 1 shows the influence of different levels of partial replacement of cement with slag (15%, 30%, and 45%) by weight on $f_{c}^{\prime }$ values at curing ages of 1, 4, 7, 14, and 28 days for three different *w_c_* ratios (0.4, 0.5 and 0.6).

The early age compressive strengths of all the slag blended mortar mixtures are significantly lower than the OPC mixes (CM-1 to CM-3). Despite this short-term negative effect, it is evident that the relative difference in strength between OPC mixes and those with GGBFS replacement reduces as the curing age increases and, for *w_c_* = 0.6, $f_{c}^{\prime }$ values for mixes with GGBFS replacement of between 15% and 45% exceed those of the OPC mix at later ages. The increase in compressive strengths arises due to the excess Calcium Silicate Hydrate (C-S-H) generated in the secondary hydration process between the SiO_2_ in the slag powder and alkaline calcium hydroxide (Ca(OH)_2_) generated in the primary hydration reaction [28]. The GGBFS reduces the enrichment of Ca(OH)_2_ crystals at the fine aggregate-cement interface and hence increases the bulk density by filling the voids and air gaps within the mixture and also reducing the overall size of the remaining Ca(OH)_2_ crystals [29]. This process only led to greater strengths compared with the OPC mix at *w_c_* = 0.6, suggesting that there is a limited supply of water in the mixture available for hydration reactions or the hydration by-product, Ca(OH)_2_, required for pozzolanic reactions when *w_c_* < 0.6 [30,31].

The $f_{c}^{\prime }$ of all slag blended mortars decreased with an increase in the GGBFS percentage in all three sets of *w_c_* ratio mixtures for curing periods up to 1 week. Between curing periods of 2 and 4 weeks, the slag blended mortars with 30% OPC replacement have the highest $f_{c}^{\prime }$ compared with replacement levels of 15% and 45%, indicating there is an optimal percentage replacement of about 30 %. Despite having more SiO_2_ contents in the slag blended mortars with 45 % OPC replacement, a reduction in OPC content leads to reduced production of Ca(OH)_2_ and vice versa.

#### 3.1.2. Influence of *w_c_* Ratio on Compressive Strength of Slag Blended Mortars

**Figure 2:**
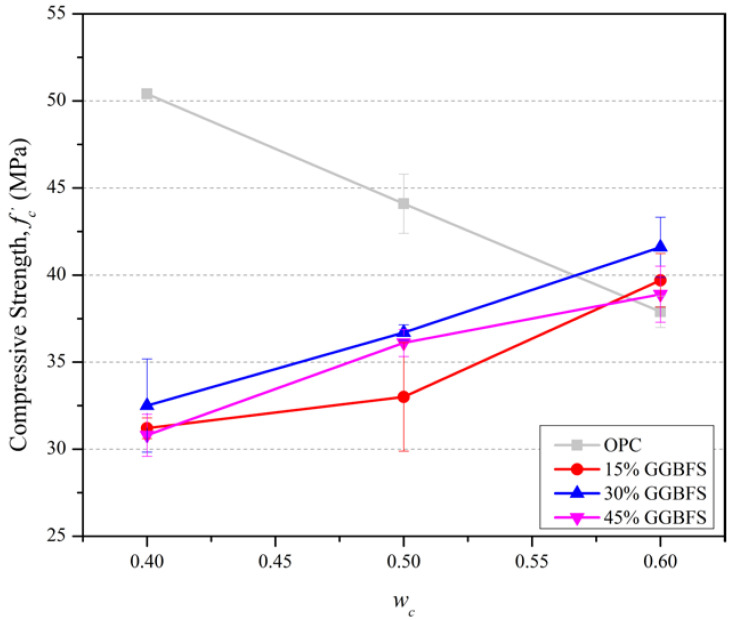
Influence of water content on $f_{c}^{\prime }$ at 28 days.

The influence of *w_c_* on $f_{c}^{\prime }$ of slag blended mortars at 28 days is presented in Figure 2. For all the slag blended mortar mixtures investigated, a consistent trend for $f_{c}^{\prime }$ at 28 days to increase with *w_c_* ratio is evident. This can be explained, in part, by the greater availability of pore space for reactant dissolution and hydration product precipitation at the higher water content [1,32].

The water in the concrete paste exists as non-evaporable water (chemically combined water), such as compound water and evaporable water in gel and capillary water. As the cement paste starts to set, the water evaporates leaving behind air voids that reduce the strength, density, and durability of cement mixtures. This process leads to the trend seen in Figure 2 of $f_{c}^{\prime }$ reducing with *w_c_* in the OPC control mixes. Incorporating fine GGBFS into OPC mixes assists in filling voids left by evaporation [1,2,3,4,33,34]. However, a large amount of GGBFS in the mixture to fill voids does not necessarily lead to greater compressive strength because of an insufficient supply of Ca(OH)_2_ generated by primary hydration of a reduced quantity of OPC. Consequently, there are excessive amounts of non-hydrated GGBFS fine powder particles. This present study found that the increase in *w_c_* generally leads to greater $f_{c}^{\prime }$ values for slag blended mortar mixtures (for the replacement percentages of 15% to 45%).

### 3.2. Ultrasonic Pulse Velocity (V_p_)

#### 3.2.1. Influence of GGBFS as a Partial Replacement of OPC on *V_p_*

**Figure 3:**
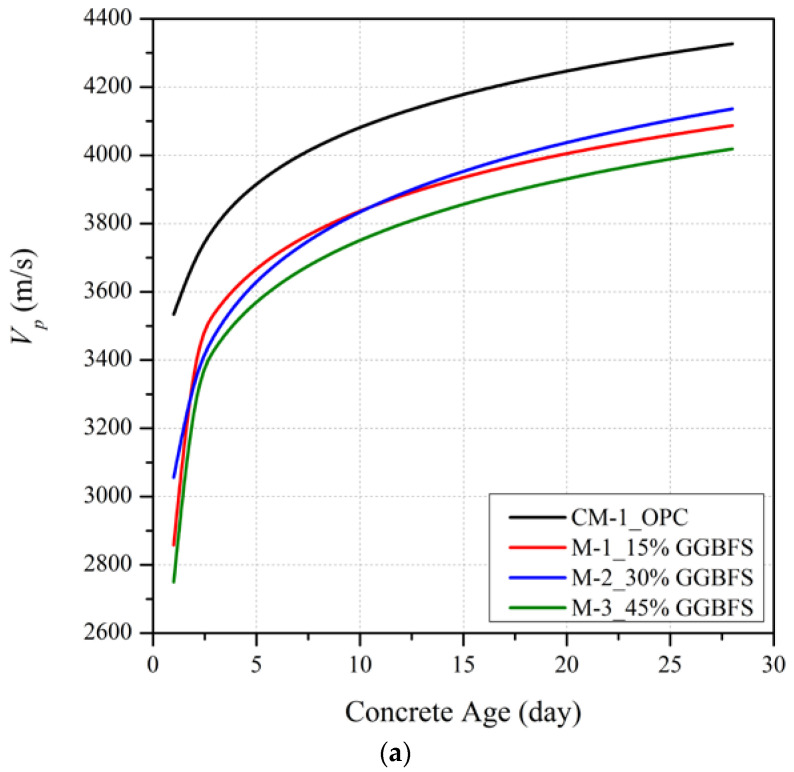

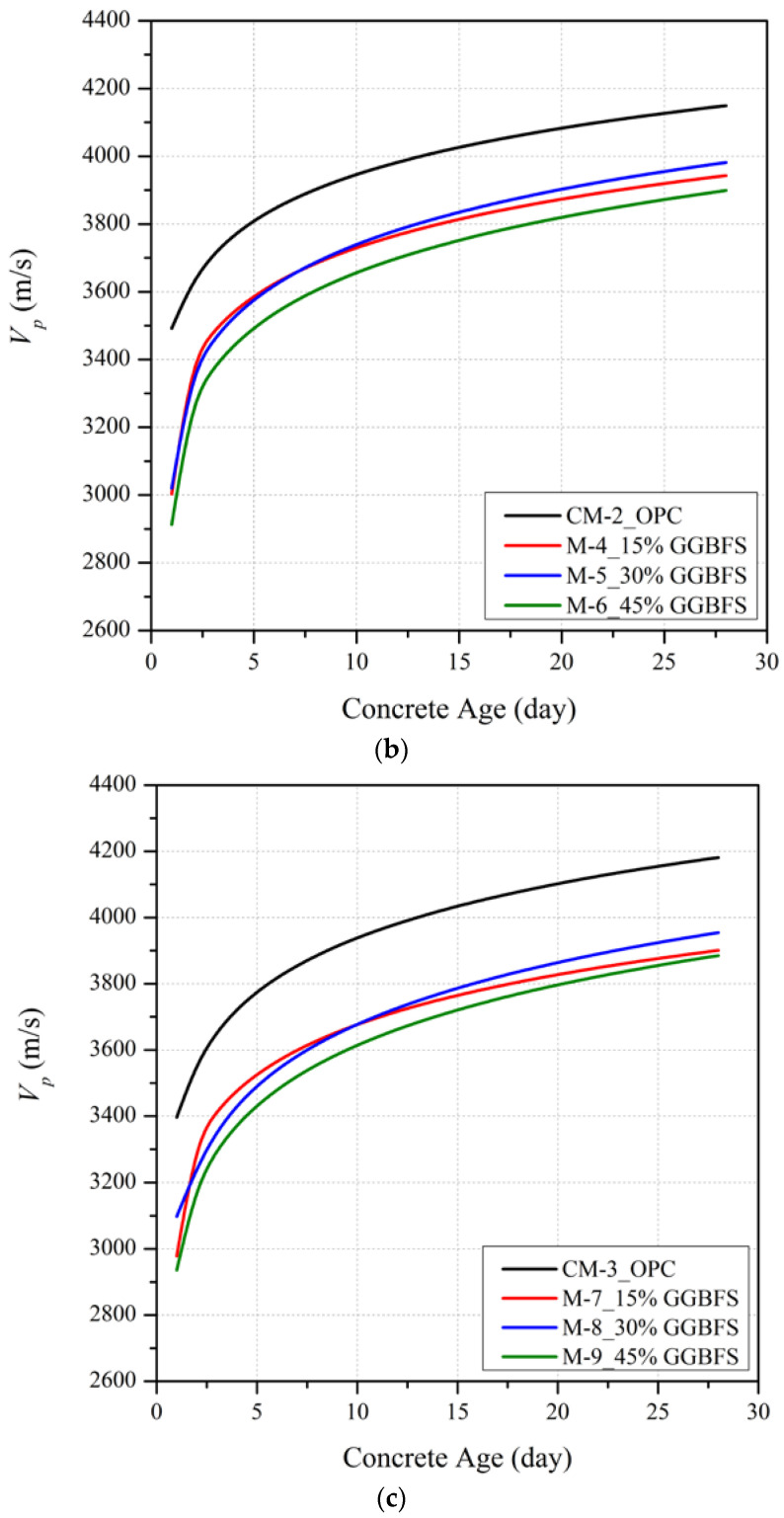
Ultrasonic pulse velocity development for slag blended mortar mixes with *w_c_* of (**a**) 0.4, (**b**) 0.5 and (**c**) 0.6.

Figure 3 illustrates the influence on *V_p_* of different levels of partial replacement of cement with GGBFS (15%, 30%, and 45%) at curing ages of 1, 4, 7, 14 and 28 days for three different *w_c_* ratios (0.4, 0.5 and 0.6). It is seen that, as for the trends with $f_{c}^{\prime }$, the mean early age *V_p_* of slag blended mortar mixtures at a constant *w_c_* is significantly less than the OPC mix. This trend reflects the slower initial hydration process as the hydration of GGBFS only begins once the alkaline level in the mixture reaches a threshold value [30,31]. From a curing period of about one week onwards, the slag blended mortar mixtures with 30% GGBFS replacement show a greater rate of *V_p_* development with time compared to the mixes with 15% and 45% GGBFS replacements. This trend is consistent with the optimal GGBFS replacement proportion of 30% for $f_{c}^{\prime }$.

Although the $f_{c}^{\prime }$ of the mix with 30% GGBFS replacement at 28 days exceeded that of the OPC mix at *w_c_* = 0.6, it is seen on Figure 3 that *V_p_* at this age is lower than the OPC mix at all *w_c_* ratios. The differences in *V_p_* are significant with shortfalls in *V_p_* at 28 days for slag blended mortars relative to the *V_p_* for OPC of 6.6%, 4.4% and 8.1% for mixes with respective *w_c_* values of 0.4, 0.5 and 0.6.

#### 3.2.2. Influence of *w_c_* on *V_p_* of Slag Blended Mortars

**Figure 4:**
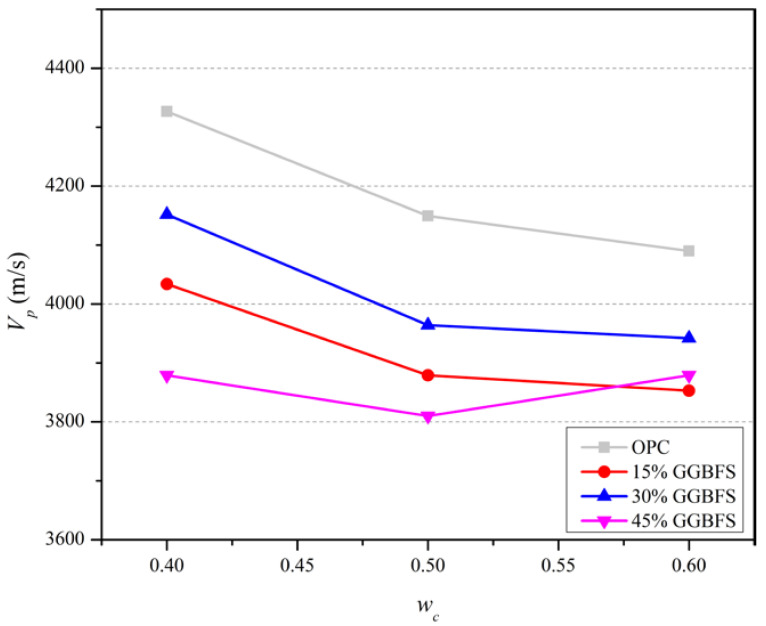
Mean value of *V_p_* at 28 days obtained by varying the amount of water content in slag blended mortar mixtures and OPC.

**Figure 5:**
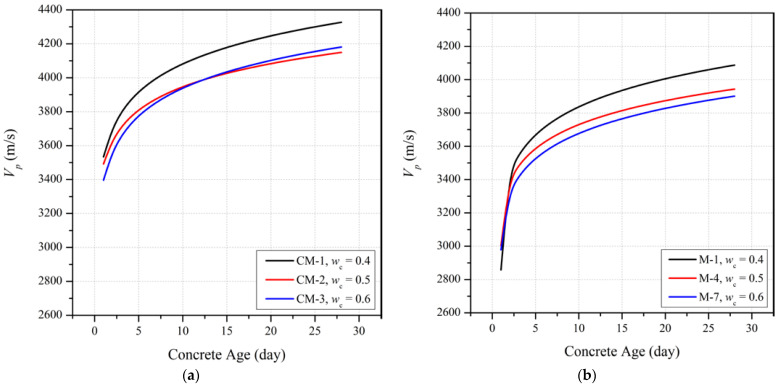

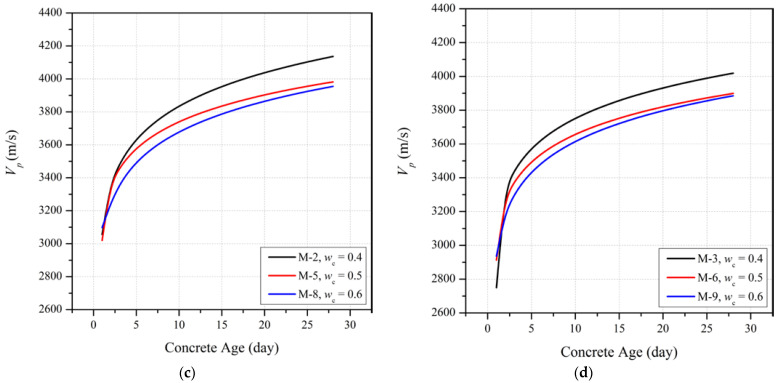
Ultrasonic pulse velocity development for (**a**) OPC mix, slag blended mortars with (**b**) 15% cement replacement, (**c**) 30% cement replacement, and (**d**) 45% cement replacement with GGBFS.

The effect of *w_c_* on the mean value of *V_p_* for all mixes at 28 days is shown in Figure 4, whilst the evolution of *V_p_* in mixes categorized by GGBFS replacement percentages are shown in Figure 5. *V_p_* decreases with increasing *w_c_* and the rate of decrease of the slag blended and OPC mortars is comparable. This trend contrasts with the variation of $f_{c}^{\prime }$ with *w_c_* shown in Figure 2 and arises because of the lower ratio of water to OPC in the slag blended mortars leading to a reduced efficiency of hydration. As the hydration kinetics of mineral cement grains and materials such as GGBFS is strongly dependent on the pore structure formation [35], excess water content increases in air void contents, the formation of micro cracks and pitting on the surface [36]. Consequently, the transmissivity of ultrasonic wave propagations through the mixture of cement, sand and GGBFS decreases with increasing *w_c_* due to wave dispersion and scattering caused by these capillary voids [37].

### 3.3. Correlation between $f_{c}^{\prime }$ and V_p_ of Slag Blended Mortars

The relationship between $f_{c}^{\prime }$ and *V_p_* for slag blended mortars is investigated using the experimental results from the current study combined with results from other tests on slag blended mortars reported in the literature [38,39,40,41]. Details from these other studies are summarised in Table 6.

The compressive strength of concrete ($f_{c}^{\prime })$ is generally estimated from the ultrasonic pulse velocity (*V_p_*) using an expression with an exponential format.

(1) $$f_{c}^{\prime }=a\cdot e^{b\cdot V_{p}}$$

where *a* and *b* are dependent on the mix composition.

The empirical coefficients (*a* and *b*) derived for each mixture with varying *w_c_* and *s* (GGBFS partial replacement percentage) values are summarised in Table 7, where $f_{c}^{\prime }$ and *V_p_* have units of MPa and m/s, respectively. It is evident from the high coefficient of determination (R^2^) in the table that Equation (1) provides a good estimate of the relationship between $f_{c}^{\prime }$ and *V_p_* but that the values of ‘*a*’ and ‘*b*’ vary with the mixture proportions.

**Figure 6:**
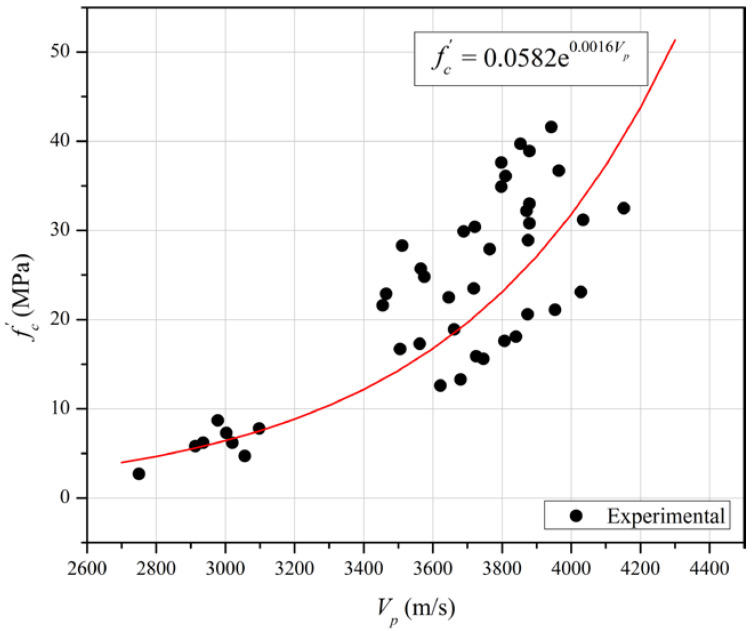
The single exponential function correlation between ultrasonic pulse velocity (*V_p_*) and compressive strength ($f_{c}^{\prime }$) from day 1 up to day 28.

The best fit single exponential trend line to all experimental data from the present study is plotted together with the data on Figure 6 and is given by:

(2) $$f_{c}^{\prime }=0.0582\cdot e^{0.0016\cdot V_{p}},\;\left(R^{2}=0.588\right)$$

**Figure 7:**
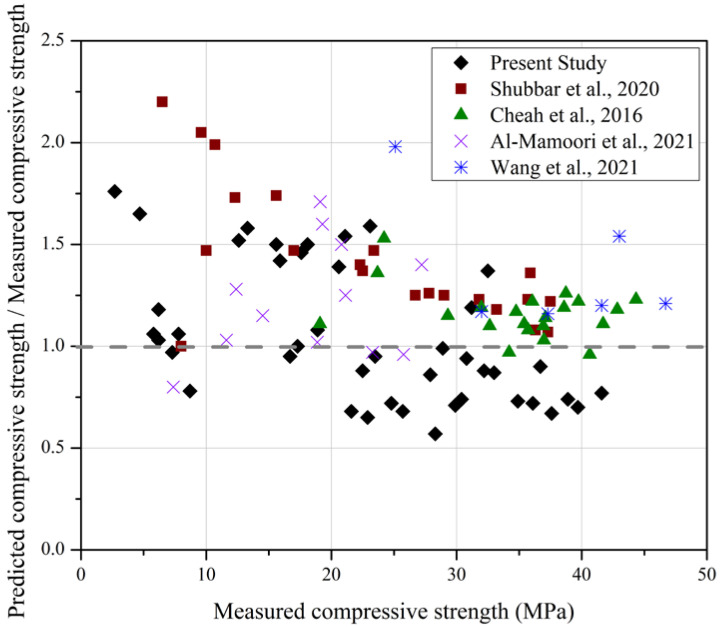
Calculated compressive strength values-to-measured compressive strength values ratio using Equation (2) [38,39,40,41].

Equation (2) has a poor R^2^ value compared to R^2^ values for the correlations for individual mixtures given in Table 6, highlighting the effect of the mix composition. A comparable low level of reliability is apparent on Figure 7, which plots the ratio of $f_{c}^{\prime }$ values calculated using Equation (2) to the measured $f_{c}^{\prime }$ values for the database of cases listed in Table 6 as well as those obtained in the current study. It is seen that the ratios of calculated to measured strengths typically vary between 1.5 and 0.66 and do not reveal a systematic dependence on the mortar mix. Calculated compressive strengths are generally higher than measured strengths for $f_{c}^{\prime }<$ 20 MPa, reflecting the effects of time and slag replacement content evident in Figure 1.

An analysis of variance (ANOVA) with a single degree of freedom (SF) and confidence interval of 95% was conducted on the data obtained from the present study to investigate the dependence of $f_{c}^{\prime }$ and *V_p_*, concrete age (*t*), water-to-cement ratio (*w_c_*) and slag replacement percentage (*s*). The results from this analysis are provided in Table 8 and indicate that *V_p_*, *t*, and *w_c_* are statically significant (with *p*-values less than 0.05) but that *s* is not significant (within the range investigated of 15% to 45%).

Regression analysis of samples with and without slag replacement gave the following best fit expressions:

(3a) $$f_{c}^{\prime }\left(\mathrm{mortar}\right)=0.3e^{0.0012V_{p}}\;\;\;\;\;\left(R^{2}=0.899\right)$$

(3b) $$f_{c}^{\prime }\left(\mathrm{mortar}\;\mathrm{with}\;\mathrm{slag}\;\mathrm{replacement}\right)=\left(\frac{0.22e^{3w_{c}}}{1+e^{-0.3t}}\right){f^{\prime }}_{c}\left(\mathrm{mortar}\right)\;\left(R^{2}=0.944\right)$$

where $f_{c}^{\prime }$ is expressed in MPa, *t* is in days, and *V_p_* is in m/s. Limits of applicability for Equation (3b) are as follows: 15% ≤ s ≤45%, 1 day ≤ t ≤ 28 days, 0.15 ≤ w_c_ ≤0.6.

The high value of R^2^ for Equation (3a) indicates that a good estimate of $f_{c}^{\prime }$(mortar) can be obtained for a given mix, knowing the value of *V_p_* alone. Equation (3b) allows determination of the strength of the mortar with slag replacement by applying a correction for the curing period and water-to-cement ratio to the compressive strength of mortar with no slag present, $f_{c}^{\prime }$(mortar). The coefficient of determination of R^2^ for Equation (3b) is significantly higher than that of Equation (2) due to the incorporation of the tendency for $f_{c}^{\prime }$ in mixes with slag replacement to reach that of $f_{c}^{\prime }$(mortar) after long curing periods and the trend of reducing strength with increasing *w_c_*.

**Figure 8:**
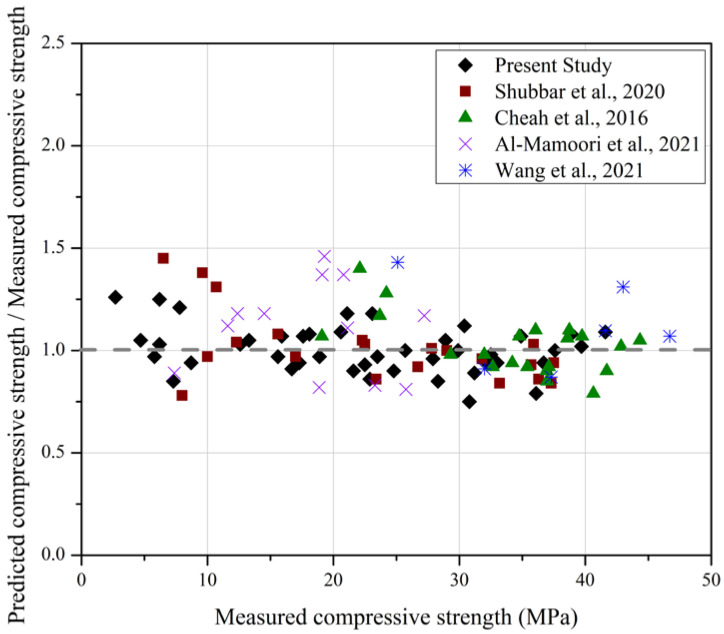
Calculated compressive strength values-to-measured compressive strength values ratio using the proposed multivariable strength prediction model—Equation (3b) [38,39,40,41].

The suitability of Equation (3) results from the previous studies summarised in Table 6 is examined in Figure 8 which presents the ratios of calculated to measured $f_{c}^{\prime }$ values determined using Equation (3). In contrast to Figure 6, calculated $f_{c}^{\prime }$ values are typically within 15% of measured values and no systematic dependence on the strength value (and the curing period by inference) is observed. The good agreement with Equation (3) also indicates that the specific $f_{c}^{\prime }$ *vs V_p_* relationship for the mortars described in Table 6 is similar to that of the mortar mix employed in the present study.

## 4. Conclusions

This study demonstrated the effect of partial replacement of cement with GGBFS in mortar and showed that:

- A slower hydration rate is observed during early ages (1-day to 14-day) but long-term strengths approach those of mortars without GGBFS. Previous research has shown that this is due to the specific characteristics of hydration kinetics of these material types.
- The compressive strength of mortars with GGBFS at a given curing period and water-to-cement ratio is relatively independent of the slag replacement percentage (*s*) for *s* values between 15% and 45%.
- The compressive strength of a given mortar mix without slag is well described by an exponential function of the ultrasonic velocity (*V_p_*).
- The exponential relationship between compressive strength and ultrasonic velocity requires the application of a correction factor in mortars with slag replacement, where the correction factor is a function of time and water-to-cement ratio.

## Figures and Tables

**Table 1:** Established empirical equations for $f_{c}^{\prime }\;$ and *V_p_*.

Author(s)	Mixture Composition	Range of Compressive Strength	%GGBFS	Expression Type	Strength Prediction Model (R2)
Bogas et al. [20]	Cement Type I 52.R and I 42.5 R with SF and FA, coarse and fine sand (2:1), gravel and light weight aggregates (<12 mm), *w_c_* = 0.3–0.65)	25–90 MPa (7–28 days)	0%	Power	$f_{c}^{\prime }={\left(\frac{V_{p}}{K_{UPV}\cdot \rho^{0.5}}\right)}^{2/3}$ (R^2^ = 0.85)
Biswas et al. [11]	Cement Type I with SF in dry densified form (2-15%), coarse aggregates (<20 mm), and fine aggregates, *w_c_* = 0.36	40–75 MPa (7–28 days)	0%	Exponential	$f_{c}^{\prime }=27.87\cdot e^{0.000198\cdot V_{p}}$ (R^2^ = 0.79)
Najim [8]	Cement Type I, mineral coarse aggregate (<20 mm), natural sand (<4.75 mm), unspecified *w_c_*	25–50 M Pa (28 days)	0%	Linear	$f_{c}^{\prime }=0.0136\cdot V_{p}-21.34$ (R^2^ = 0.70)
Trtnik et al. [17]	CEM II/A-S 42.5 R, CEM I 52.5 R, CEM I 42.5 N, and CEM I 42.5 N SR, with crushed limestone and rounded limestone	0–50 MPa (1–7 days)	0%	Exponential	$f_{c}^{\prime }=0.0854\cdot e^{1.2882\cdot V_{p}}$ (R^2^ = 0.64)
Demirboga et al. [3]	ASTM Type 1 cement with FA and BFS (50-70%), natural aggregates (<16 mm), and w/b = 0.35	2–55 MPa (3–120 days)	50–70%	Exponential	$f_{c}^{\prime }=0.0049\cdot e^{0.0021\cdot V_{p}}$ (R^2^ = 0.96)
Le et al. [18]	ASTM Type I OPC, with coarse aggregates (<20 mm), fine aggregates (<5 mm), FA, GGBFS, and sugarcane bagasse ash (SBA), w/b = 0.45	20–35 MPa (7–91 days)	0–60%	Exponential	$f_{c}^{\prime }=1.82\cdot e^{0.0007\cdot V_{p}}$ (R^2^ = 0.94)
Turkmen et al. [19]	ASTM Type 1 OPC, with coarse aggregate (<16 mm), fine aggregate (<4 mm), and cement substitution using either NZ, or BFS, or both, w/b = 0.4	5–50 MPa (3–90 days)	0–30%	Exponential	$f_{c}^{\prime }=0.0301\cdot e^{0.0017\cdot V_{p}}$ (R^2^ = 0.94)

%GGBFS—partial replacement percentage of cement using GGBFS, BFS—blast furnace slag, FA—fly ash, SF—silica fume and NZ—natural zeolite.

**Table 2:** Chemical composition of cement, silica sand and ground granulated blast furnace slag (major components listed).

Cement		AFS 45/50 Silica Sand		GGBFS	
CaO	63.4%	SiO_2_	99.9%	S	0.4%
SiO_2_	20.1%	Fe_2_O_3_	0.01%	SO_3_	2.4%
Al_2_O_3_	4.6%	Al_2_O_3_	0.02%	MgO	5.7%
Fe_2_O_3_	2.8%	CaO	0.00%	Al_3_O_3_	12.6%
SO_3_	2.7%	MgO	0.00%	FeO	0.8%
MgO	1.3%	Na_2_O	0.00%	MnO	0.1%
Na_2_O	0.6%	K_2_O	0.00%	Cl	0.01%
Total chloride	0.02%	TiO_2_	0.03%	Insoluble residue content	0.2%
-	-	MnO	<0.001%	-	-

AFS—American Foundry Society.

**Table 3:** Physical properties of cement, silica sand and ground granulated blast furnace slag.

Cement		AFS 45/50 Silica Sand		GGBFS	
Specific Gravity	3.15	Loss on ignition	0.01	Specific gravity	3.0–3.2
Fineness Index	390 m^2^/kg	Water content (at 105 °C)	<0.001	Relative Water Requirement	103%
Normal Consistency	27%	AFS fineness number	47.5	Loss on Ignition	0.20%
Setting Time Initial	120 min	-	-	Temperature Rise	18.8 °C
Setting Time Final	210 min	-	-	Fineness (passing 45 μm)	98%
Soundness	2 mm	-	-	-	-
Loss on Ignition	3.80%	-	-	-	-
Fineness (passing 45 μm)	95.30%	-	-	-	-

**Table 4:** Natural fine sand aggregate distribution.

Sieve Size (μm)	Percentage Passing
1180	100
600	91
300	14.8
150	3.1
75	0

**Table 5:** Mix proportions by weight.

Mix	OPC	Sand	Water	GGBFS	Cement Proportion	GGBFS Proportion	Superplasticizer (mL/kg)
CM-1	1	2 *	0.4	0	100%	0%	2.55
CM-2	1	2	0.5	0	100%	0%	1.48
CM-3	1	2	0.6	0	100%	0%	0
M-1	0.85	2	0.4	0.15	85%	15%	2.94
M-2	0.70	2	0.4	0.30	70%	30%	2.86
M-3	0.55	2	0.4	0.45	55%	45%	3.64
M-4	0.85	2	0.5	0.15	85%	15%	1.76
M-5	0.70	2	0.5	0.30	70%	30%	1.07
M-6	0.55	2	0.5	0.45	55%	45%	0.91
M-7	0.85	2	0.6	0.15	85%	15%	0
M-8	0.70	2	0.6	0.30	70%	30%	0
M-9	0.55	2	0.6	0.45	55%	45%	0

* Note deviation from value of 2.75 recommended in ASTM C109/C109M.

**Table 6:** Previous studies on slag blended mortars.

Concrete Mix Composition	Range of Com-pressive Strength, $f_{c}^{\prime }$ (MPa)	GGBFS Partial Replacement, *s* (%)	Specimen Shape and Size	Reference
Ordinary Portland cement mortar—partial replacement with GGBFS (10–40%) and CKD (5–25%), fine aggregates (<3.15 mm), *w_c_* = 0.4	Normal strength mortar (5–38 MPa at 1–28 days)	0–40%	100 mm cubes	Shubbar et al. [40]
Portland limestone cement mortar—partial cement replacement with intergrinded GGBFS (0–80%), and PFA (0–20%), fine aggregates (<5 mm), *w_c_* = 0.45	Normal strength mortar (19–44 MPa at 7–28 days)	0–80%	100 mm cubes	Cheah et al. [39]
CEM-II / A / LL 32.5-N cement mortar—partial cement replacement with GGBFS (0–35%) and PFA (0–35%), fine aggregates (<4.76 mm), *w_c_* = 0.4	Normal strength mortar (8–32 MPa at 7–28 days)	0–35%	100 mm cubes	Al-Mamoori et al. [38]
ASTM Type 1 cement mortar—partial cement replacement with GGBFS (0–20%) and SSRS (5–20%), fine aggregates (<4.75 mm), *w_c_* = 0.5	High strength mortar (25–63 MPa at 3–56 days)	0–20%	50 mm cubes	Wang et al. [41]

CKD—cement kiln dust; PFA—pulverized fly ash; SSRS—stainless steel reduced slag.

**Table 7:** Regression coefficients corresponding to each slag blended mixture.

a	b	*w_c_*	s (%GGBFS)	R^2^
0.2114	0.0012	0.4	15	0.939
0.0243	0.0017		30	0.995
0.0124	0.0019		45	0.968
0.0518	0.0016	0.5	15	0.985
0.0191	0.0019		30	0.976
0.0190	0.0019		45	0.989
0.0521	0.0017	0.6	15	0.993
0.0232	0.0019		30	0.903
0.0210	0.0020		45	0.976
0.2982	0.0012	0.4–0.6	0	0.900

**Table 8:** Analysis of variance among variables on $f_{c}^{\prime }$.

Source of Variation	SS	MS	F-Value	*p*-Value
*t* (days)	5749.14	5749.14	51.22	0
*V_p_* (m/s)	398,365,031.60	398,365,031.60	6002.57	0
*s* (%)	137.82	137.82	0.66	0.42
*w_c_*	17,487.02	17,487.02	268.309	0

SS—sum of the square variation; MS—mean squares.

## Data Availability

All the data is available within the manuscript.
